# EMPress Enables Tree-Guided, Interactive, and Exploratory Analyses of Multi-omic Data Sets

**DOI:** 10.1128/mSystems.01216-20

**Published:** 2021-03-16

**Authors:** Kalen Cantrell, Marcus W. Fedarko, Gibraan Rahman, Daniel McDonald, Yimeng Yang, Thant Zaw, Antonio Gonzalez, Stefan Janssen, Mehrbod Estaki, Niina Haiminen, Kristen L. Beck, Qiyun Zhu, Erfan Sayyari, James T. Morton, George Armstrong, Anupriya Tripathi, Julia M. Gauglitz, Clarisse Marotz, Nathaniel L. Matteson, Cameron Martino, Jon G. Sanders, Anna Paola Carrieri, Se Jin Song, Austin D. Swafford, Pieter C. Dorrestein, Kristian G. Andersen, Laxmi Parida, Ho-Cheol Kim, Yoshiki Vázquez-Baeza, Rob Knight

**Affiliations:** a Department of Computer Science, Jacobs School of Engineering, University of California, San Diego, California, USA; b Center for Microbiome Innovation, Jacobs School of Engineering, University of California, San Diego, California, USA; c Bioinformatics and Systems Biology Program, University of California, San Diego, California, USA; d Department of Pediatrics, School of Medicine, University of California, San Diego, California, USA; e Algorithmic Bioinformatics, Justus Liebig University, Giessen, Germany; f IBM T. J. Watson Research Center, Yorktown Heights, New York, USA; g IBM Almaden Research Center, San Jose, California, USA; h Department of Electrical and Computer Engineering, University of California, San Diego, California, USA; i Center for Computational Biology, Flatiron Institute, Simons Foundation, New York, New York, USA; j Scripps Institution of Oceanography, University of California, San Diego, California, USA; k Department of Bioengineering, University of California, San Diego, California, USA; l Scripps Research Institute, San Diego, California, USA; m IBM Research, The Hartree Centre, Daresbury, United Kingdom; n Collaborative Mass Spectrometry Innovation Center, Skaggs School of Pharmacy and Pharmaceutical Sciences, University of California, San Diego, California, USA; o Cornell Institute for Host-Microbe Interaction and Disease, Cornell University, Ithaca, New York, USA; University of Waterloo

**Keywords:** bioinformatics, microbial ecology

## Abstract

Standard workflows for analyzing microbiomes often include the creation and curation of phylogenetic trees. Here we present EMPress, an interactive web tool for visualizing trees in the context of microbiome, metabolome, and other community data scalable to trees with well over 500,000 nodes. EMPress provides novel functionality—including ordination integration and animations—alongside many standard tree visualization features and thus simplifies exploratory analyses of many forms of ‘omic data.

**IMPORTANCE** Phylogenetic trees are integral data structures for the analysis of microbial communities. Recent work has also shown the utility of trees constructed from certain metabolomic data sets, further highlighting their importance in microbiome research. The ever-growing scale of modern microbiome surveys has led to numerous challenges in visualizing these data. In this paper we used five diverse data sets to showcase the versatility and scalability of EMPress, an interactive web visualization tool. EMPress addresses the growing need for exploratory analysis tools that can accommodate large, complex multi-omic data sets.

## INTRODUCTION

The increased availability of sequencing technologies and automation of molecular methods have enabled studies of unprecedented scale (1), prompting the creation of tools better suited to store, analyze (2), and visualize (3) studies of this magnitude. Many of these tools, including for example UniFrac (4), phylofactor (5), PhILR (6), and Gneiss (7), make use of tree structures in some way: often these structures are phylogenetic trees detailing the evolutionary relationships among features in a data set, although this category also includes general dendrograms that organize features in a hierarchical structure (e.g., clustering of mass spectra) (8). The challenge of enabling fully interactive analyses stems from the disconnect between tools that focus on features (for example, microbial relative abundances) and tools that focus on samples (for example, alpha diversity distributions). In addition, few tools can interactively integrate multiple representations of the data side-by-side (9) while scaling to display large data sets. We view this as a key unresolved challenge for the field: to contextualize community-level patterns (groupings of samples) together with feature-level structure, i.e., which features lead to the groupings explained in a given sample set.

Although many useful phylogenetic visualization and analysis tools are available, few focus on community analysis tasks. The current state of the art includes specialized tools like Anvi’o (10), which consolidates a large collection of methods for sequence-based analysis and visualization of metagenomic assembled-genomes, pangenomes, and proteins (among many other data types). The state of the art also includes more general-purpose tree visualization tools like PHYLOViZ (9), SigTree (11), and iTOL (12) (among many others). Although tree structures are usually stored in standard file formats like Newick, the data accompanying these trees—for example, tip-level taxonomic classifications or other metadata values—are less standardized and sometimes require the onerous creation of configuration files. Furthermore, some types of exploratory analyses are not easily possible: for example, ordination plots computed from UniFrac (4) distances (or other phylogenetically informed distances) are often used to visualize sample clustering patterns in microbiome studies. However, interpreting the patterns in these plots—and determining which features influence the separation of certain groups of samples—is not always straightforward. While biplots can improve legibility by showing information about influential features alongside samples, the phylogenetic relationships of these features are not always obvious.

To address these and other outstanding gaps in the state of the art, we introduce EMPress (https://github.com/biocore/empress), an open-source (BSD 3-clause), interactive and scalable phylogenetic tree viewer accessible as a QIIME 2 (2) plugin or as a standalone Python program. EMPress is built around the high-performance balanced parentheses tree data structure (13) and uses a hardware-accelerated WebGL-based rendering engine that allows EMPress to visualize trees with hundreds of thousands of nodes from within a laptop’s web browser (see Materials and Methods). EMPress visualizations can be created solely from a tree, or users can optionally provide additional metadata files and a feature table to augment the visualization. Additionally, through integration with the widely-used EMPeror software (3), EMPress can simultaneously visualize a study’s phylogenetic tree alongside an ordination plot of the samples in the data set (in what we colloquially term an “EMPire plot”). User actions in one visualization, such as selecting a group of samples in the ordination, update the other (in this case, highlighting the portions of the tree corresponding to these samples), providing context that would not be easily accessible with independent visualizations. This tight integration between displays streamlines several use-cases elaborated below that previously required manual investigation or writing custom scripts.

## RESULTS

**FIG 1:**
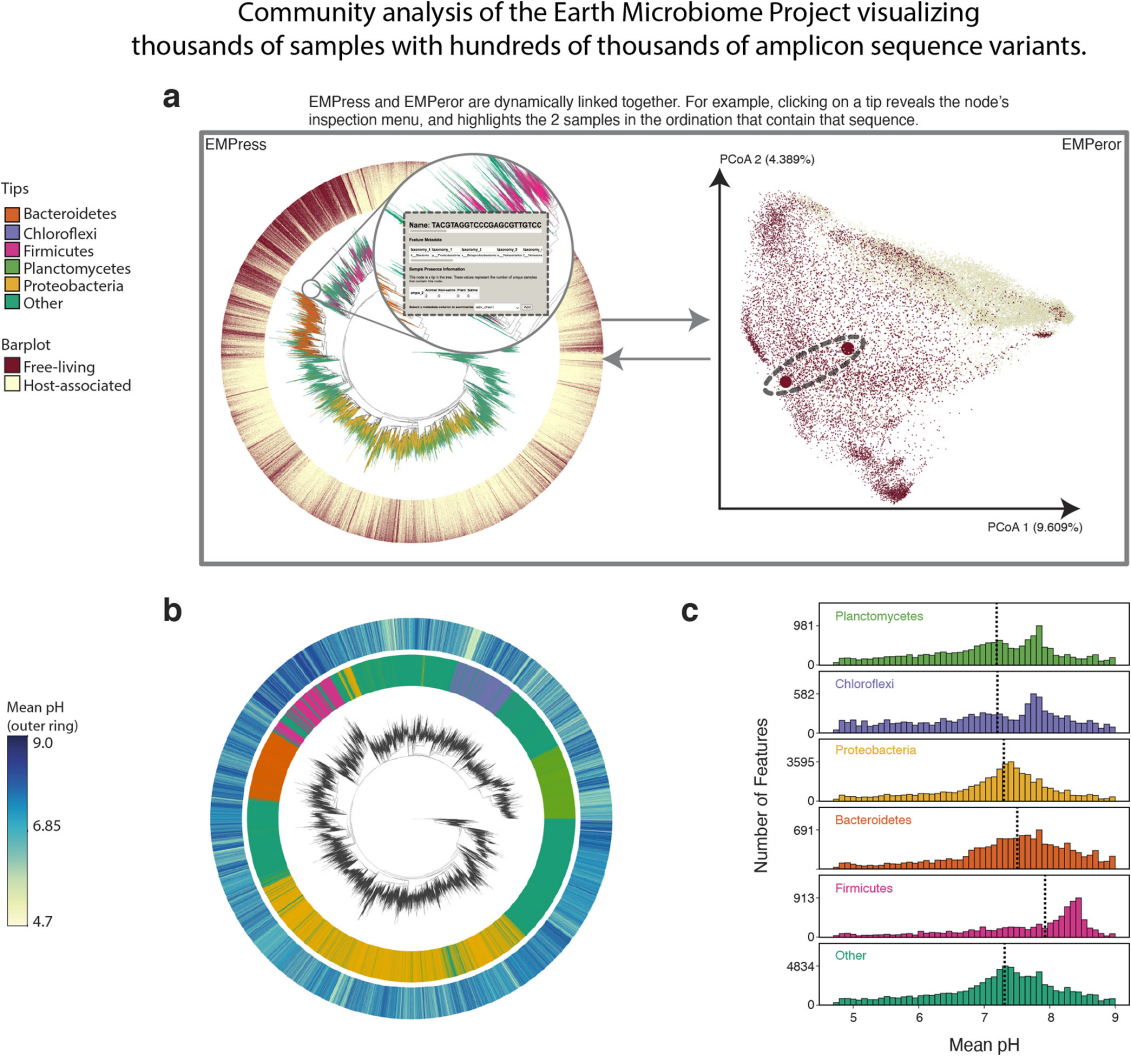
Earth Microbiome Project paired phylogenetic tree (including 756,377 nodes) and unweighted UniFrac ordination (including 26,035 samples). (a) Graphical depiction of EMPress’ unified interface with fragment insertion tree (left) and unweighted UniFrac sample ordination (right). Tips are colored by their phylum-level taxonomic assignment; the barplot layer is a stacked barplot describing the proportions of samples containing each tip summarized by level 1 of the EMP ontology. Inset shows summarized sample information for a selected feature. The ordination highlights the two samples containing the tip selected in the tree enlarged to show their location. (b) Subset of EMP samples with pH information: the inner barplot ring shows the phylum-level taxonomic assignment, and the outer barplot ring represents the mean pH of all the samples where each tip was observed. (c) pH distributions summarized by phylum-level assignment with median pH indicated by dotted lines. Interactive figures can be accessed at https://github.com/knightlab-analyses/empress-analyses.

Rather than providing a programmatic interface for the procedural generation of styled phylogenetic trees (14, 15; FigTree [http://tree.bio.ed.ac.uk/software/figtree/]), EMPress provides an interactive environment to support exploratory feature- and sample-level tree-based analyses. One of the ways EMPress stands out is in its scalability in comparison to other web-based tree viewers: iTOL (12) claims trees with more than 10,000 tips to be “very large” (https://itol.embl.de/help.cgi), while EMPress readily supports trees with over hundreds of thousands of tips, as shown in Fig. 1. Many visualization customization options available in EMPeror (3), iTOL (12), and Anvi’o (10) are immediately accessible in EMPress’ interface. Continuous metadata associated with the tips of the tree can be visualized as barplots with a color gradient and/or by mapping each value to the height of each bar. Similarly, categorical sample metadata information can be visualized using a stacked barplot showing—for each tip—the proportion of samples containing that tip stratified by category. These options are available in EMPress’ user interface, based on the data provided by the user to EMPress when creating a visualization, and—providing data files are stored in an accepted format—do not require programming or the creation of configuration files.

EMPress also aids interpretation of ordination plots by optionally providing a unified interface where the tree and ordination visualizations are displayed side-by-side and “linked” through sample and feature identifiers (16). This combination of EMPress and EMPeror (3) allows for many novel exploratory data analysis tasks. For example, selecting a group of samples in the ordination highlights nodes in the tree present in those samples and vice versa (see Materials and Methods). This integration extends to biplots: clicking feature arrows in the ordination highlights the corresponding node in the tree. Lastly, EMPress supports the visualization of longitudinal studies by simultaneously showing tree nodes unique to groups of samples at each individual time point during an EMPeror animation (see Materials and Methods).

### Scalability: visualizing data from the Earth Microbiome Project.

Using the first data release of the Earth Microbiome Project (EMP), we demonstrate EMPress’ scalability by rendering a 26,035-sample ordination and a 756,377-node tree (Fig. 1a). We also demonstrate EMPress’ ability to annotate large tree visualizations with categorical “feature” (i.e., corresponding to nodes in the tree) metadata: to visualize the relative proportions of taxonomic groups at the phylum level, we use EMPress’ feature metadata coloring to color tips in the tree by their phylum-level taxonomic classifications (see Materials and Methods). We extend this further by demonstrating EMPress’ ability to visualize sample-level categorical metadata: we add a barplot layer showing, for each tip in the tree, the proportions of samples containing each tip summarized by level 1 of the EMP ontology (“Free-living” and “Host-associated”).

The paired “EMPire plot” visualizations supported by EMPress’ integration with EMPeror allow users to click on a tip in the tree (in EMPress) and view the samples that contain that feature in the ordination (in EMPeror). Clicking on an internal node in the tree functions similarly, showing all samples that contain any of the descendant tips of this node. These actions, and other functionality unique to these paired visualizations, are especially useful when analyzing data sets with outliers or mislabeled metadata. Figure 1a shows an example of this functionality in practice, in which the samples in which a tip in the tree is present are highlighted dynamically in an ordination.

EMPress’ barplots can also be used to summarize environmental metadata: as a demonstration of this, Fig. 1b shows the subset of samples (4,002) with recorded pH information and a barplot layer with the mean pH where each feature was found. The barplot reveals a relatively dark section near many tips classified in the phylum *Firmicutes*; in concert with histograms showing mean pH for each phylum (Fig. 1c), we can confirm that *Firmicutes*-classified sequences are more commonly found in higher-pH environments. These and the other observations highlighted here indicate the utility of EMPress for exploratory analyses of large, complex data sets.

### Versatility: visualizing diverse types of data.

EMPress—both in the visualization of tree structures and in the visualization of various types of metadata alongside these structures—can be applied to many types of “‘omic” data sets. To illustrate this versatility, we reanalyzed a COVID-19 metatranscriptome sequencing data set (17), a liquid chromatography-mass spectrometry (LC-MS) untargeted metabolomic food-associated data set (8), and a 16S rRNA gene sequencing oral microbiome data set (18). Despite the vastly different natures of these data sets, EMPress provides meaningful functionality for their analysis and visualization. Movie S1 in the supplemental material also shows a longitudinal exploratory analysis using EMPress and EMPeror representing a subset of SARS-CoV-2 genome data from GISAID. This paired visualization emphasizes the relationships in time and space among “community samples” and the convergence of locales in the United States with the outbreak in Italy (see Materials and Methods). The interactive nature of EMPress allows rapid visualization of strains observed in a collection of samples from different geographical locations.

10.1128/mSystems.01216-20.3 MOVIE S1 Longitudinal community analysis of SARS-CoV-2 using phylogenetically informed animations. Each tip in the tree represents a SARS-CoV-2 genome. Each sphere in the ordination represents the genomes collected over a 7-day window. This movie showcases some analytical capabilities available in EMPress. Download Movie S1, MOV file, 11.4 MB. Copyright © 2021 Cantrell et al. 2021 Cantrell et al. https://creativecommons.org/licenses/by/4.0/ This content is distributed under the terms of the Creative Commons Attribution 4.0 International license.

**FIG 2:**
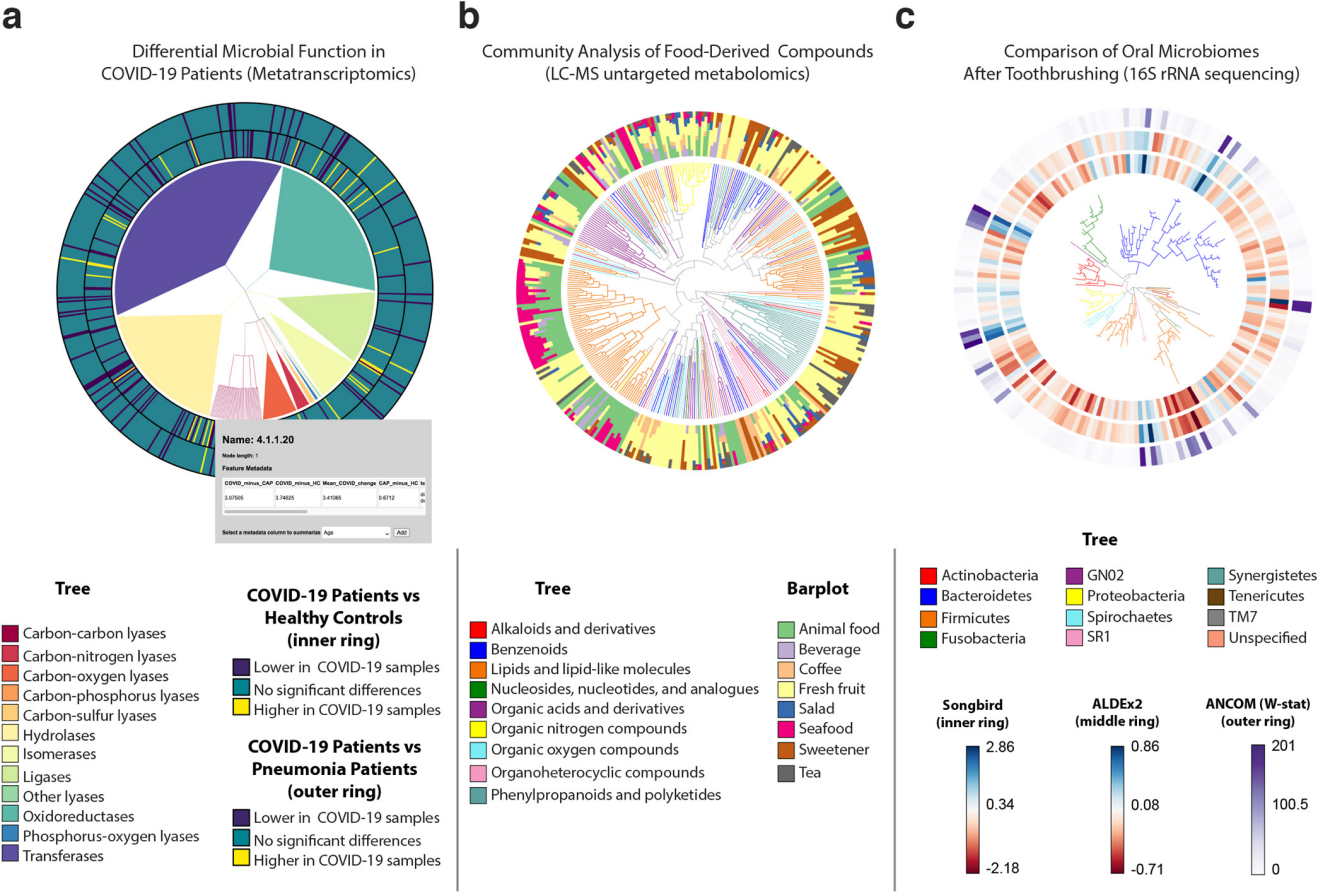
EMPress is a versatile exploratory analysis tool adaptable to various ‘omics data types. (a) RoDEO differential abundance of microbial functions from metatranscriptomic sequencing of COVID-19 patients (*n* = 8) versus community-acquired pneumonia patients (*n* = 25) and versus healthy control subjects (*n* = 20). The tree represents the four-level hierarchy of the KEGG enzyme codes. The barplot depicts significantly differentially abundant features (*P* < 0.05) in COVID-19 patients. Clicking on a tip produces a pop-up insert tabulating the name of the feature, its hierarchical ranks, and any feature annotations. (b) Global FoodOmics Project LC-MS data. Stacked barplots indicate the proportions of samples (*n* = 70) (stratified by food) containing the tips in an LC-MS Qemistree of food-associated compounds, with tip nodes colored by their chemical superclass. (c) *De novo* tree constructed from 16S rRNA sequencing data from 32 oral microbiome samples. Samples were taken before (*n* = 16) and after (*n* = 16) subjects (*n* = 10) brushed their teeth; each barplot layer represents a different differential abundance method’s measure of change between before- and after-brushing samples. The innermost layer shows estimated log-fold changes produced by Songbird, the middle layer shows effect sizes produced by ALDEx2, and the outermost layer shows the W-statistic values produced by ANCOM (see Materials and Methods). The tree is colored by tip nodes’ phylum-level taxonomic classifications. Interactive figures can be accessed at https://github.com/knightlab-analyses/empress-analyses.

Figure 2a showcases EMPress’ ability to identify feature clusters that are differentially abundant in COVID-19 patients compared to community-acquired pneumonia patients and healthy controls (17). Clades showing KEGG enzyme code (EC) (19) annotations are collapsed at level two except for lyases, highlighting feature 4.1.1.20 (carboxy-lyase diaminopimelate decarboxylase) that was more abundant in COVID-19 here and in an independent metaproteomic analysis of COVID-19 respiratory microbiomes (20).

Recent developments in cheminformatics have enabled the analysis and visualization of small molecules in the context of a cladogram (8). Using a tree that links molecules by their structural relatedness, we analyzed untargeted LC-MS/MS data from 70 food samples (see Materials and Methods). With EMPress’ sample metadata barplots, we can inspect the relationship between chemical annotations and food types. Figure 2b shows a tree where each tip is colored by its chemical superclass and where barplots show the proportion of samples in the study containing each compound by food type. This representation reveals a clade of lipids and lipid-like molecules that are well represented in animal food types and seafoods. In contrast, salads and fruits are broadly spread throughout the cladogram.

Lastly, in Fig. 2c, we compare three differential abundance methods in an oral microbiome data set (18) as separate barplot layers on a tree. This data set includes samples (*n* = 32) taken before and after subjects brushed their teeth (see Materials and Methods). As observed across the three differential abundance tools’ outputs, all methods agree broadly on which features are particularly “differential” (for example, a group of sequences classified in the phylum *Firmicutes* in the bottom right of the tree; see Materials and Methods), although there are discrepancies due to different methods’ assumptions and biases.

## DISCUSSION

### Future work on visualization integration.

By providing an intuitive interface supporting both categorically new and established functionality, EMPress complements and extends the available range of tree visualization software. EMPress can perform community analyses across distinct “’omics” types, as demonstrated here. Moving forward, facilitating the integration of multiple orthogonal views of a data set at a more generalized framework level (for example, using QIIME 2’s [2] visualization API) will be important as data sets continue to grow in complexity, size, and heterogeneity.

### Validating visual observations and aiding reproducibility.

It is important to note that making visual observations using EMPress does not eliminate the need for providing statistical support. For example, various layout and branch length options available in EMPress can drastically affect the perceived size of a clade or taxonomic group. We therefore recommend that users remain careful of the need to validate their claims, in order to ensure that conclusions drawn are not solely visual artifacts. In the context of microbial ecology studies, tools like Phylofactor, Gneiss, and PhILR can complement observations made with EMPress well.

Similarly, although the various exploratory (*post hoc*) analyses shown in this paper are simplified by EMPress, they are not a substitute for sound hypothesis-driven science and should not be presented as such (21). Exploratory analyses, when documented transparently, can be useful to the scientific community (21); our hope is that, by providing a tool that simplifies these analyses of complex data, EMPress can fulfill a legitimate scientific need. One way we believe EMPress helps fulfill this need is through the inherent shareability of its outputs (see Materials and Methods). This shareability simplifies the process of reproducing a visualization in EMPress, as well as the interactive exploration of alternative representations of a data set. As an example of this, we have provided QZV files for Fig. 1 and 2 on GitHub (see Materials and Methods), and we encourage readers to reproduce these figures for themselves. We acknowledge that “reproducibility” of this kind is limited to the files the user provides when creating an EMPress visualization—and since the same data and same methods are used in the reproduction as in the original visualization, this therefore does not necessarily add evidence that conclusions derived from the visualization are completely correct (22). However, we contend that it can still be beneficial for readers and authors alike, and we hope that by simplifying the sharing of EMPress visualizations we can encourage researchers to share their visualizations and make clear the exploratory nature of their work.

### Tree shearing in the context of community data.

In order to aid in the analysis of community data, EMPress—when a feature table is provided—by default shears the tree to include only tips that are found in the feature table. This preprocessing step visually emphasizes samples spanning only a small number of tips within larger reference trees. Since this option may not always be desired—if, for example, the focus of an analysis is to compare novel diversity to a reference database—this option can be disabled from the command line.

## MATERIALS AND METHODS

All EMPress plots in this paper were visualized and stylized in Safari (14.0) or Google Chrome (85.0.4183.121) using a MacBook Pro (15-inch, 2017) with a 2.9-GHz Quad-Core Intel Core i7 7820QM, 16 GB of RAM, a Radeon Pro 560 4 GB, and Intel HD Graphics 630 1,536-MB graphics processor. Data, analyses, and steps to reproduce the figures in this paper can be found at https://github.com/knightlab-analyses/empress-analyses. Due to file size restrictions on GitHub, some files are downloaded by executing the Jupyter notebook associated with each figure.

### Earth Microbiome Project.

The EMP release 1 (1) table, tree, and metadata were used to generate the visualization (ftp://ftp.microbio.me/emp/release1). The original feature table was subset to remove sterile water blanks and mock community samples. The table contains the taxonomic assignments used to annotate tips. For ease of visualization, only the top 5 most abundant phylum annotations in the data set were kept while microbial features annotated with any other phylum (or unspecified) were categorized as “Other.” A distance matrix was generated by computing the unweighted UniFrac distances between samples (4, 23) that was then used to generate the principal-coordinate plot.

A subset of the feature table was generated by extracting all samples in the middle 90% of the pH range (4.7 to 9) to remove outliers. Samples without a valid pH value were removed. For each remaining feature, the number of samples (log_10_) in which this feature occurred and the mean pH of those samples were calculated and saved as a feature metadata file passed into EMPress. Calculations were performed using NumPy v1.18.1 (24) and Pandas v0.25.3 (25, 26). Distributions of pH were plotted using matplotlib v3.1.3 (27) and seaborn v0.10.0 (28).

### COVID-19 metatranscriptome data set.

The COVID-19 bronchoalveolar lavage fluid metatranscriptome sequencing data (17) consists of COVID-19 (*n* = 8), community-acquired pneumonia (*n* = 25), and healthy control (*n* = 20) samples. The tree for this data set corresponds to the KEGG enzyme code (EC) (19) hierarchy. Sequencing reads were processed and annotated with EC feature labels using PRROMenade (29, 30) with a database of bacterial and viral protein domains from the IBM Functional Genomics Platform (31), as previously reported (30). Differential abundance per feature was determined by performing a Kolmogorov-Smirnov test on average RoDEO-processed (30, 32) values per sample. A cutoff (*P* < 0.05) was applied to focus the visualization on the features that are significantly more abundant or less abundant in COVID-19 patients than in healthy controls and/or community-acquired pneumonia samples.

### Global FoodOmics data set.

The untargeted metabolomics data set was generated using a quadrupole time of flight (QTOF) mass spectrometer in positive ionization mode (Bruker). The samples presented in this data set were processed using Qemistree version 2020.1.1+14.g1b4edb4 running in QIIME 2 version 2019.7. For ease of interpretation, the data set was subset to keep features with a superclass assignment and keep samples with a *common meal type* classification. The tip barplots show the proportion of samples where each small molecule is present summarized by *meal type*.

### Differential abundance comparison of oral microbiomes.

The oral microbiome 16S rRNA sequencing data used in reference 18 was revisualized for Fig. 2c. This data set comprises *n* = 32 samples total, taken before and after subjects brushed their teeth. Some paired samples were taken more than once from the same subject; in total, these 32 samples were contributed by 10 unique subjects.

The sequences in this data set were processed (in July 2018) using Deblur v1.0.4 (33) through q2-deblur in QIIME 2 2018.6 (2). Taxonomic classifications were assigned (in August 2018) using q2-feature-classifier’s (34) classify-sklearn method (35), using the Greengenes reference database (36), also in QIIME 2 2018.6. The table provided lacks provenance information due to not being stored as a QIIME 2 artifact, but since its features are a subset of those in the sequences file—and since the lowest number of samples that a feature within it is present in is 6—it was likely filtered at some point. To construct a rooted tree from the sequences in this data set (in September 2020), we used QIIME 2 2019.10’s qiime phylogeny align-to-tree-mafft-fasttree pipeline (37–39).

In reference 18, three differential abundance tools were run on this data set, using the “brushing_event” metadata field (indicating before/after toothbrushing status) as the sole field across which to identify differentially abundant features. The three differential abundance tools used in reference 18 and visualized in Fig. 2c are Songbird (18), ALDEx2 (40), and ANCOM (41). Songbird’s column of feature differentials (describing the estimated log-fold changes of each feature between the “after” and “before” brushing states) is shown as the innermost barplot layer in Fig. 2c; ALDEx2’s per-feature effect size is shown as the middle layer; and ANCOM’s per-feature W-statistic is shown as the outermost layer. For both Songbird and ALDEx2 results, higher values indicate association with before-brushing samples (i.e., features that decreased most from toothbrushing, for example, secondary metabolizers present on the outer layers of dental plaque biofilms such as *Haemophilus*) while lower values indicate association with after-brushing samples (i.e., features that decreased least from toothbrushing, for example, primary metabolizers such as *Actinomyces* that are rooted at the base of the biofilm) (18). ANCOM’s W-statistic corresponds to the number of log-ratio hypothesis tests in which a given feature was found to be differentially abundant between before- and after-brushing samples (41) (https://forum.qiime2.org/t/1844/10). Since Songbird and ALDEx2’s results include directionality between before and after brushing, they are shown in Fig. 2c with a “diverging” color map; ANCOM’s W-statistic does not include this information and is therefore shown with a “sequential” color map (see Fig. S1 in the supplemental material).

10.1128/mSystems.01216-20.1 FIG S1 Scatterplot comparing the three differential abundance methods’ results shown in Fig. 2c. The *x* axis and *y* axis represent the Songbird differentials and ALDEx2 effect sizes for the features in the data set, while each feature point is colored by its ANCOM W-statistic. This demonstrates that our starting interpretation of how these results relate—i.e., that Songbird and ALDEx2’s results have similar “directionality” between the before- and after-toothbrushing states and that ANCOM’s W-statistic lacks this same directionality—is reasonable. The nine features for which no Songbird differentials were available are omitted from this plot. This scatterplot was produced using matplotlib (50). Download FIG S1, PDF file, 0.06 MB. Copyright © 2021 Cantrell et al. 2021 Cantrell et al. https://creativecommons.org/licenses/by/4.0/ This content is distributed under the terms of the Creative Commons Attribution 4.0 International license.

We note that the Songbird results from reference 18 did not include differentials for nine features in the data set; this may have been due to software bugs or other unknown factors in the data analysis, since (although Songbird does filter out features present in less than a given number of samples) these absent features are present in the same number of samples as other features which were included in the Songbird differentials. For the sake of simplicity here, and since the purpose of this subfigure is primarily to demonstrate the utility of EMPress in the context of existing data, we simply reused the data from reference 18, filtering these nine features out of the data set before constructing and visualizing the tree.

### Animated analysis of SARS-CoV-2.

The GISAID (42) SARS-CoV-2 genome alignment and genome metadata were obtained on 21 September 2020. Sequences were converted to DNA and subset to the set of sequences associated with Italy, Madrid, King County, San Diego, Brooklyn, Queens, and Manhattan. Highly gapped and high-entropy positions in the alignment were filtered using q2-alignment (2020.6; default parameters). A tree was estimated using FastTree (38) (v2.1.10 compiled with double precision support; default options except -fastest) and subsequently rooted using midpoint rooting as implemented by q2-phylogeny (2020.6; default parameters).

Separately, a sliding window procedure was developed to assess the observed SARS-CoV-2 genomes within a given time period within a geographic location. To do so, the metadata were partitioned into the respective locations (note that the three New York boroughs were treated as New York) and ordered by the genome date information. A sliding window width of 7 days was used, and a sample was retained only if five or more strains were observed within a window. These windows were then aggregated into a BIOM table (43) with the GISAID strain identifier on one axis and a “community sample” identifier on the other. Unweighted UniFrac (q2-diversity 2020.6 [2, 23]) was then computed over these samples followed by a principal-coordinate analysis. The tree and ordination were visualized with a development version of EMPress (version 0.3.0-dev), and therefore, the visualization shown in the video may look slightly different from more recent versions of EMPress.

### Implementation details.

EMPress is implemented as a QIIME 2 plugin (or standalone Python program, usable outside QIIME 2) capable of generating HTML documents with a self-contained visualization user interface. The code-base is composed of a Python component and a JavaScript component. The Python code-base is responsible for data validation, preprocessing, filtering, and formatting. User interaction, rendering, and figure generation are all handled by the JavaScript code-base. In both cases, we rely on the balanced parentheses data structure (13) to rapidly operate on the tree structures.

EMPress’ Python code-base currently uses NumPy (24), SciPy (44), Pandas (25, 26), Click (https://palletsprojects.com/p/click/), Jinja2 (https://jinja.palletsprojects.com/), scikit-bio (http://scikit-bio.org), the BIOM format (43), iow (https://github.com/wasade/improved-octo-waddle) (13), and EMPeror (3). The JavaScript code-base uses Chroma.js (https://gka.github.io/chroma.js/), FileSaver.js (https://github.com/eligrey/FileSaver.js/), glMatrix (http://glmatrix.net/), jQuery (https://jquery.com/), Require.js (https://requirejs.org/), Spectrum (https://bgrins.github.io/spectrum/), and Underscore.js (https://underscorejs.org/). For testing and linting, EMPress’ Python code-base uses flake8 (https://flake8.pycqa.org/en/latest/) and nose (https://nose.readthedocs.io/), and EMPress’ JavaScript code-base uses QUnit (https://qunitjs.com/), qunit-puppeteer (https://github.com/davidtaylorhq/qunit-puppeteer/), jshint (https://jshint.com/about/), and Prettier (https://prettier.io/).

As of writing, EMPress supports drawing trees using three standard layout algorithms (“rectangular,” “circular,” and “unrooted”), coloring the tree using sample and feature metadata, collapsing clades based on common metadata values, adding tip-aligned barplots for sample and feature metadata, and summarizing tips’ presence within sample groups using interactive node selections. This is in addition to integration with EMPeror, described further below, as well as various other visualization options.

EMPress’ “unrooted” layout algorithm is translated from code from Gneiss (7), which was in turn adapted from PyCogent (45), and is an implementation of the equal-angle algorithm described in reference 46. EMPress’ “rectangular” and “circular” layout algorithms are adapted from code from TopiaryExplorer (47) and resemble the rooted tree drawing algorithms described in reference 46. EMPress also includes the ability to reorder sibling clades in the rectangular and circular layouts by the number of tips contained within each clade; this functionality was inspired by iTOL’s (12) “leaf sorting” option and uses tree traversal code adapted from scikit-bio (http://scikit-bio.org).

In order to integrate EMPress and EMPeror, we link together events triggered by each of the applications by inserting “callback” code that can be executed in one application when a given event occurs. These events notify each tool that a particular action needs to take place, and if needed, what data should be used in this context. For example, when a user selects a group of samples in EMPeror, the “select” event is triggered with a collection of sample objects. EMPress responds to this event by searching for the tips in the tree corresponding to features contained within these samples and updates the color according to the object’s attributes. The subscription mechanism also enables users to select a node in EMPress to highlight the samples containing this node or one of its descendant tips in EMPeror, link biplot (48) arrows in EMPeror to nodes in the tree, highlight groups by double-clicking a category in EMPeror’s color legend, and synchronize animated ordinations (49) by coloring the tree according to the current frame on screen.

### Sharing EMPress visualizations.

When used as a QIIME 2 (2) plugin, EMPress generates visualizations in the QZV format (which can be viewed using https://view.qiime2.org, in addition to other methods); when used outside QIIME 2, EMPress creates visualizations as a directory (containing an HTML file that can be opened to show the visualization). In either case, an EMPress visualization is easily shareable with a wide audience of users who may not have EMPress or QIIME 2 installed, for example, via uploading the visualization file(s) to GitHub or by hosting this file(s) on any other website. We note that the ability to share visualizations is not unique to EMPress and is also inherent to other QIIME 2 (2) plugins and in other tree visualization tools like iTOL (12).

10.1128/mSystems.01216-20.2 TABLE S1 We gratefully acknowledge the authors and originating and submitting laboratories of the sequences from GISAID’s database on which the analysis in Movie S1 is based. The list is detailed below. Download Table S1, XLSX file, 0.2 MB. Copyright © 2021 Cantrell et al. 2021 Cantrell et al. https://creativecommons.org/licenses/by/4.0/ This content is distributed under the terms of the Creative Commons Attribution 4.0 International license.
