# Supplementary figures and images for: EMPress Enables Tree-Guided, Interactive, and Exploratory Analyses of Multi-omic Data Sets
Source: mSystems. 2021 Mar 16;6(2):e01216-20. doi: 10.1128/mSystems.01216-20 (PMC8546999; doi:10.1128/mSystems.01216-20)

# Differential abundance methods: an alternate representation

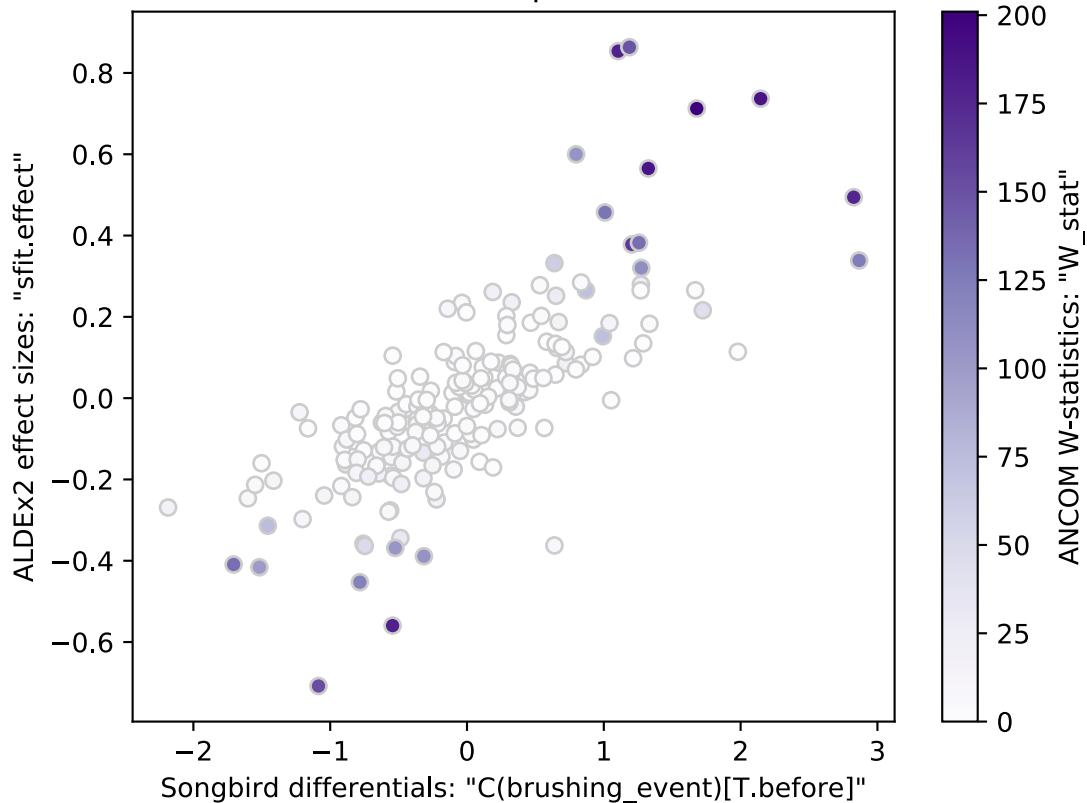

Supplement: FIG S1 [file msystems.01216-20-sf001.pdf]
